# A new model for lipid monolayer and bilayers based on thermodynamics of irreversible processes

**DOI:** 10.1371/journal.pone.0212269

**Published:** 2019-04-04

**Authors:** O. A. Pinto, E. A. Disalvo

**Affiliations:** 1 Instituto de Bionanotecnología del NOA (INBIONATEC), Universidad Nacional de Santiago de Estero (UNSE- CONICET), Villa el Zanjón, Santiago del Estero, Argentina; 2 Laboratorio de Biointerfases y Sistemas Biomiméticos, Centro de Investigaciones en Biofisica Aplicada y Alimentos (CIBAAL) (UNSE-CONICET), Villa el Zanjón, Santiago del Estero, Argentina; Bose Institute, INDIA

## Abstract

Lipid monolayers are used as experimental model systems to study the physical chemical properties of biomembranes. With this purpose, surface pressure/area per molecule isotherms provide a way to obtain information on packing and compressibility properties of the lipids. These isotherms have been interpreted considering the monolayer as a two dimensional ideal or van der Waals gas without contact with the water phase. These modelistic approaches do not fit the experimental results. Based on Thermodynamics of Irreversible Processes (TIP), the expansion/compression process is interpreted in terms of coupled phenomena between area changes and water fluxes between a bidimensional solution of hydrated head groups in the monolayer and the bulk solution. The formalism obtained can reproduce satisfactorily the surface pressure/area per lipid isotherms of monolayer in different states and also can explain the area expansion and compression produced in particles enclosed by bilayers during osmotic fluxes. This novel approach gives relevance to the lipid-water interaction in restricted media near the membrane and provides a formalism to understand the thermodynamic and kinetic response of biointerphases to biological effectors.

## Introduction

The structural backbone of the biological membranes is the lipid bilayer which consists of lipids in contact with the aqueous environment stabilized by a combination of hydrophobic and hydrophilic interactions [1]. The interplay of these forces are thought to give living cells a way to control their responses to changes in the environment to adapt and survive. Due to the complexity of living systems, lipid monolayers and bilayers of known composition have been extensively used as simplified model systems to study the lipid membrane properties in order to make more comprehensible and predictable the behavior of biological systems. In this regard, thermodynamics as a general law seems to be the adequate conceptual frame.

**Fig 1:**
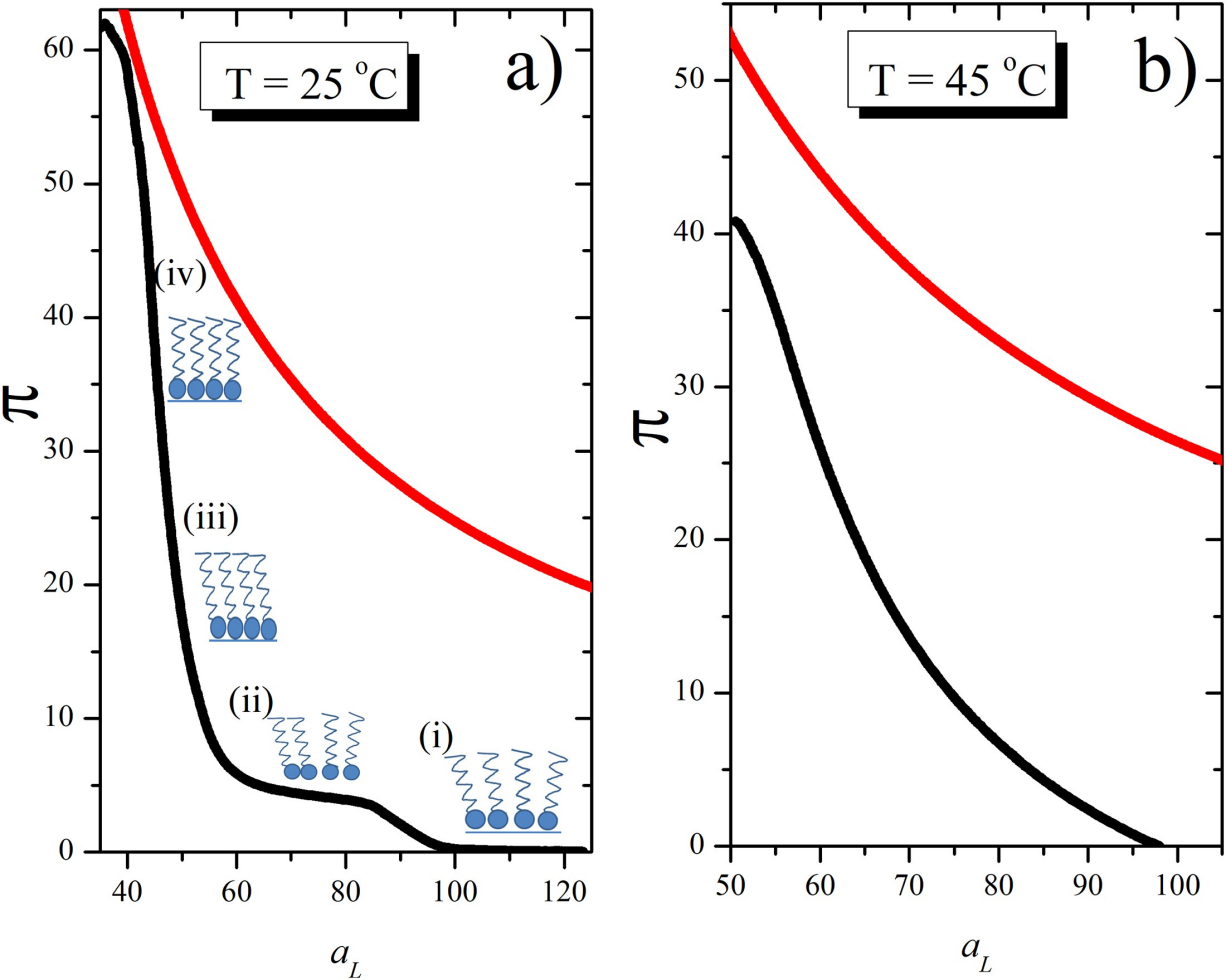
Surface pressure versus area per molecule for an ideal gas model. Experimental (black line) and calculated (red line) π/A per lipid curves for DPPC considering the lipid monolayer as an ideal gas dispersed on an (inert) water surface, according to equation $\pi =\frac{RT}{a_{L}}$ at 25°C (a) and 45°C (b) Experimental data of area per lipid were taken from bibliography and surface pressure calculated by means of the ideal gas equation.

One point within this goal is to correlate the equivalence of physical chemical properties between monolayers and bilayers as model systems. In this regard, Feng et at [2] considered that in plane lipid stacking is different in monolayers and bilayers and care should be taken in order to compare both systems. This might be possible provided that the tail-tail interaction of the opposing monolayers affects the packing of the surface groups. In contrast, comparison of changes of turbidity in liposome dispersions and dipole potential in monolayers, determined by the hydration forces, gave similar behaviors at the phase transition in bilayers and monolayers which was taken as a probe of similar properties of both model systems [3]. A point that has obscured the possibility of comparison under thermodynamic rules is that modelling for each experimental set up has been treated independently. On one hand, volume changes in cells has been mimicked by closed lipid vesicles in which water exchange has been measured at expense of changes in osmotic pressure [4,5]. On the other hand, lipid monolayers have been treated as a gaseous bidimensional system spread on water ignoring water exchange. In the swelling/shrinkage processes, vesicles are supposed to expand or contract due to water influx/efflux respectively, although the surface tension cannot be controlled. In contrast, monolayers provide a way to measure membrane interfacial tension controlling the lateral pressure or the area per lipid but water fluxes cannot be measured “in situ” [6]. Although a phenomenological analysis of permeation in terms of tension illustrates the mechanochemical properties of the lipid membrane, a proposal with a thermodynamic base for both systems that could give sustain to biological responses of membranes has not been reported. The consideration that water is a constituent of membrane structure that confers specific thermodynamic surface properties due to the surface tension of water seems to be a way for a novel proposal in this regard. The surface tension of water (γ^o^) decreases when an amphiphilic compound is spread on the air-water interphase and the difference is expressed as the surface pressure, *π* = *γ*^o^ − *γ*. Surface pressure can be increased by decreasing the area of the interphase available for a given lipid amount with a mobile barrier. In this condition, surface pressure/area per molecule isotherms are obtained. This procedure is done in a Langmuir balance at different temperatures [7, 8]. Typical curves below and above the critical point as that shown in Fig 1a) and 1b) respectively (black lines) are obtained. The experimental π/A curves resembles the PV isotherms of real gases [1, 2, 7, 9] in which departures from ideality is considered by introducing parameters related to the surface pressure and the finite area of the lipids as in a bidimensional van der Walls isotherm (Fig A in S1 File). A similar state to the ideal one should be achieved at temperatures above the critical point, i.e. the phase transition temperature. In this condition, the experimental curves as that shown in Fig 1(black line) are obtained, in which no coexistence is observed.

The consideration of the monolayer as a real gas implies the interactions between lipids themselves and that the lipids are non-punctual particles, i.e. they have an excluded volume that is manifested in a critical area upon compression to reach the collapse pressure [10–12].

These two deviations from the ideal behavior can, in principle, be expressed as the term a/A^2^ (pressure correction considering the intermolecular interactions) and the coarea b (area correction).

**Fig 2:**
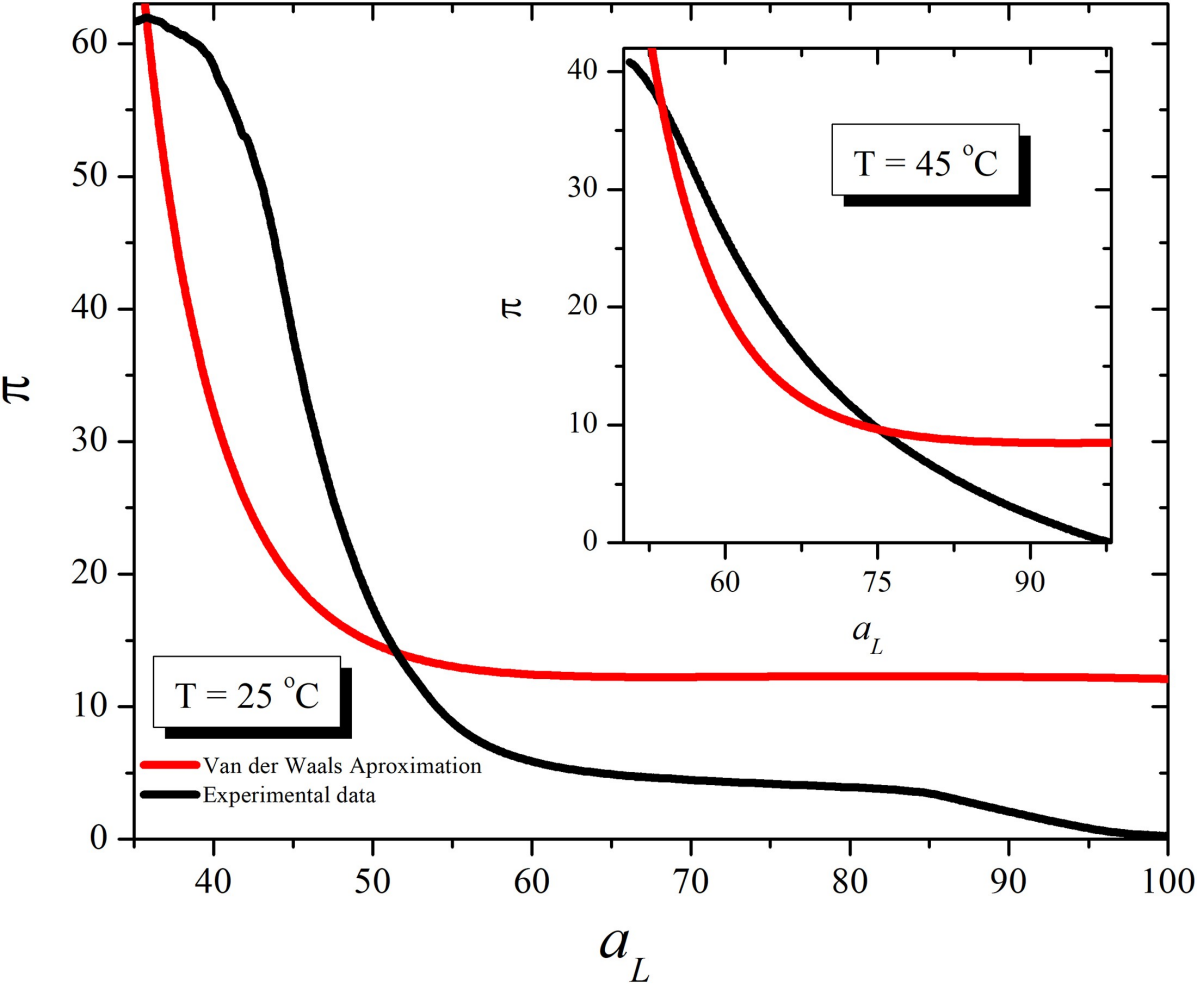
Surface pressure versus area per molecule for monolayer considered as van der Waals bidimensional gas. Same data of experimental curves as in Fig 1 (black line) are compared with a calculated π/A per lipid curves for DPPC considering the lipid monolayer as a van der Waals gas dispersed on an (inert) water surface at 25°C(a) and 45°C(b). The procedure was similar to that described in Fig 1.

Again when experimental area data was plotted considering a van der Waals approach given by (*π* + *a*/*A*^2^)(*A* − *b*) = *nRT*, the best values *a* and *b* at 25°C and 45°C give curves with a strong departure from the experimental curves (Fig 2a and 2b). Moreover, at high temperatures and large areas, where it is expected that the gas system would behave similar to an ideal one, experimental data does not overlap with the fitted curve. Hence, van der Waals equation is not suitable to explain the surface pressure/area isotherm of a lipid monolayer even in the case in which weak or none lipid-lipid interaction occurs. This means that other forces should be considered in order to explain the behavior of lipid monolayers.

**Fig 3:**
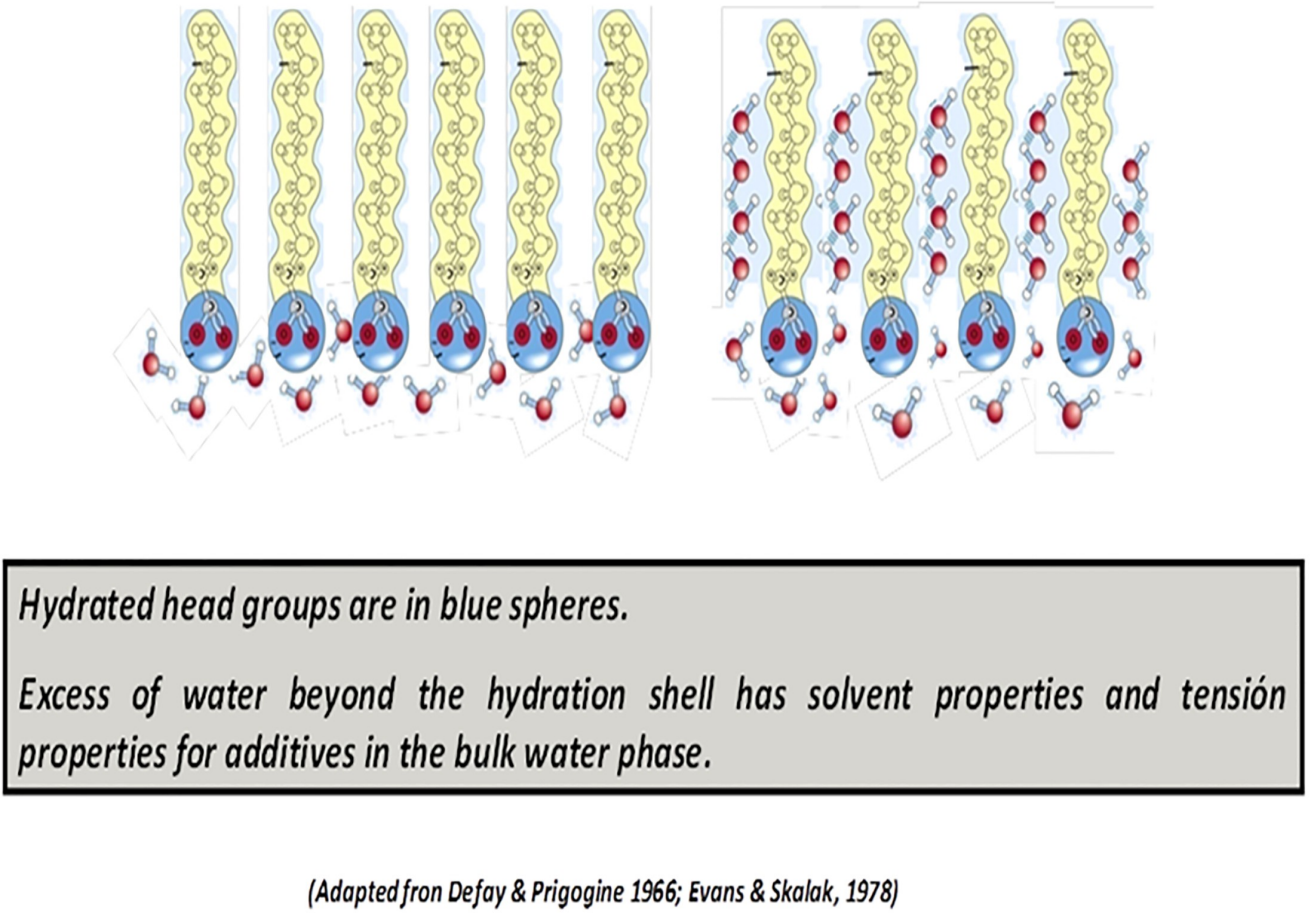
Schematic representation of lipid interphase showing lipids and its hydration shells.

In our view, if lipids are considered to be spread as a gas on an inert solvent -as a gas in vacuum interactions of lipids with the water surface as a support are neglected. However, this omission is contrary to the definition of surface pressure given above, since this is defined as the difference between the surface tension of pure water and the surface tension of the surface with the amphiphilic. In this context, lipid-lipid interactions and coarea needs redefinition in realistic terms in the light of abundant experimental information related to hydration of lipids, area per lipid and thickness of the monolayer/bilayer, excluded volume, water polarization [13–15]. Usually, the lipid-lipid interaction is explained in terms of chain-chain interaction giving place to different organizations (Fig B in S1 File) [16]. However, it is implicit and known that lipid interacts also along the hydrated head groups, which in some kind of lipids determine the packing by H bond interactions [17]. The area per lipid found in monolayers and bilayers are consistent with the inclusion of a hydration shell around the polar head groups that are composed by phosphocholine residues and carbonyls. Thus, lipid-lipid interactions can be composed by head-head repulsions and acyl chain attractions determining an excluded volume [10, 15, 18]. In this regard, lipids form a hydration shell with a finite volume that constitute a region of a define thickness where head groups are imbibed in water. This region is defined as an interphase, i.e. as a new phase having properties different that the two phases between which it is interposed (tail region and bulk water). Thus, it is considered as a surface bidimensional solution of hydrated polar groups in which thermodynamic considerations can be applied [19, 20]. In Fig 3 a graphical description is presented.

Head groups conform an excluded volume per se and another due to water molecules hydrating them (the hydration shell, blue circles). At high pressures (low areas) no water beyond this hydration shell is expected and the limit of area per group is achieved before the collapse. At lower pressures, water beyond the lipid hydration shell is found, thus the interphase can be visualized as hydrated head groups dissolved in water (Fig C in S1 File). Moreover, it has been shown recently that the nanomechanical properties of monolayers are sustained by considering the hydration state of the phospholipids [21].

The serious departures from the experimental curves of simulated π/A curves have been related to the lack of evaluation of intermolecular forces [22]. This is to say that lipid-lipid interaction should be redefined in terms of other interactions. An immediate reply to this flaw is that the supporting aqueous solution of the monolayers and the dispersing media forming bilayers should be carefully considered in terms of water-lipid interactions. The modern aspects of membrane biophysics reached by molecular dynamics simulations (CHARMS, AMBER, GROMACS) includes water as a necessary component for stabilization. In this regard, main force field between lipids is introduced associated to a given model for water. These are different according to the physical chemical properties ascribed to each model (H bonds and dipoles). This point is a strong argument in the sense that the thermodynamics of monolayer/bilayer systems should include water-lipid interaction and consequently its repercussion on membrane behavior [22, 23].

Considering the above scheme, following the approach of Defay and Prigogine, [19] the lipid monolayer is defined as a bidimensional solution of hydrated head groups immersed in water at the surface as ions in a solution [19]. In this view, when solutes dissolve in the interphase (i.e. the bidimensional solution of head groups) the chemical potential of water decreases and thus, water from the bulk phase will be displaced to the interphase increasing the surface pressure (at constant area) or increasing the area at constant pressure. Thus, the interphase monolayer is considered as an open system in which water can be exchanged from and to the bulk phase as in an osmotic process. As stated before, hydration also play a role in the nanomechanical properties of monolayers [21]. In conclusion, the evidences that the lipid monolayer interacts with the water phase cannot be ignored in a rigorous thermodynamic treatment of the lipid membrane. The new thermodynamic frame should be consistent to link monolayer and bilayer systems.

The water contribution, considered previously by several authors through studies on osmotic stress, has not been taken into account to interpret the π/A per lipid curves of lipid monolayers of Fig 1 [13–15]. These works illustrate how water forms structured oriented regions by contact with the lipids that determines several of its mechanical and electrical properties. In order to introduce a thermodynamic general formalism valid for monolayers and bilayers, these types of interactions should be taken into account. For this, it is necessary to redefine the monolayer/bilayer as a real system in which the lipids are non-punctual particles (i.e. with a finite and define volume consisting in lipids and its hydration shells) and that lipids spread on water has to displace against a frictional force on the surface. This means that the monolayer or the bilayer as thermodynamic systems should consider that the water phase affects the membrane properties. This new approach can be analyzed considering coupled phenomena within the scope of Thermodynamic of Irreversible Process (TIP)[24]. For these reasons, the purpose of the present work is to develop a general thermodynamic formalism of a lipid monolayer π/A per lipid curves in order to put into relevance the crossed interaction of lipids and water in the compression/expansion processes suitable to compare with the changes occurring in bilayer systems under osmotic gradients. It is expected that this evaluation could give a more rigorous insight of the physical chemistry of monolayers that can be extended to other membrane systems, such as bilayers.

## Thermodynamics of irreversible processes (TIP) applied to monolayer/bilayer systems

The process to obtain a surface pressure vs area/lipid curve is to move a barrier on the air/water interface seeded with a given amount of lipids at controlled temperature. A particular point in this procedure is that the movement of the barrier should be slow enough to allow to the system to equilibrate at each point. It is similar to the process of heating in a DSC assay in which heating and cooling should be done at an extremely low rate. This observation makes clear that in a compression/ expansion process a dissipative phenomenon occurs. It is possible to think this process in two types of systems. One of them is a closed one in which the components are only lipids spread on the surface. No contact of the lipids with the subphase is allowed and no exchange of lipids takes place. Thus, the barrier is pushed and a mechanical work is exerted. The equilibrium is reached when the external pressure applied to the barrier equals the internal pressure of the monolayer due to collisions of molecules with the barrier as happens with volume work in a gas at constant temperature. However, another experimental fact must be explained. If a constant amount of lipids is held by the barrier at constant pressure, the addition of more lipids would promote the expansion of the monolayer. This process can also be explained by arguing that more particles are in the gas monolayer needing more space. Then, there are more particles in the system which expands against a constant external pressure. Following the same logic, to achieve the initial state by applying an external pressure, a reduction in the number of lipids molecules in the same amount as those added is required. This is contradictory to the definition of closed system.

A more complex situation may be analyzed. Let us assume a monolayer formed by a given amount of lipids in the surface at constant lateral pressure on the barrier. Let us consider that lipids cannot abandon the surface due to its extremely low solubility in water. In this condition, a solute is added to the subphase and an increase in the surface pressure is observed. It may be argued that the solute has dissolved in the monolayer increasing the packing and thus, the surface pressure. By definition, the surface pressure increase is due to a decrease in the surface tension of the surface. Therefore, free spaces should be available in the monolayer in order to include the solute. If this is the case, the surface pressure would have a limit in the size of the solute, i.e. there would be solutes large enough that cannot fit into the free spaces between lipids. Thus, large particles as proteins would not affect the surface pressure of lipid monolayers which is certainly not the case. The increase in area to promote a change in surface pressure by a peptide or a protein is equivalent to no more than 3–5 water molecules [20].

Of course, it could be argued that some specific amino acid enters the spaces and not the entire protein in which case the affinity would be directly given by the molecular size of the solute and not by its chemical structure and independent of the process of transference from an aqueous media to the membrane. Rigorously, the partition of the solute in the membrane is driven by the difference in chemical potential of the solute in water and the solute in the membrane taken a as solvent.

If the monolayer is considered as a bidimensional gas, water does not take part of the equilibrium and thus, the pressure on the barrier would be only counterbalanced by the interaction between the lipid (without water). Under this approach the properties of lipid monolayers cannot be matched with those of bilayers because water can permeate it alone when vesicles are subjected to hypotonic or hypertonic osmotic stress producing swelling or shrinkage respectively. The gas model does not apply in interphases of bilayers.

TIP approach allows to analyzing the behavior of the systems by considering all the forces that are acting on them. The general fluxes (*J*_*i*_) driven by all forces acting on the system (*X*_*i*_) are given by

$$J_{i}=\sum l_{jj}X_{i}$$ (1)

where the *l*_*jj*_ are the phenomenological coefficients. For instance, in a system consisting by a semipermeable membrane separating two compartments containing equal volumes of a solution of a solute that does not permeate, pressure applied to one of the compartments, would displace water to the other compartment producing a dilution of the solution and increasing the concentration in the compartment at which the pressure (P) is applied.

The work done on the system is

$$W=f\Delta l=\left(\frac{f}{A}\right)\Delta lA=P\Delta V$$ (2)

where *Δl* is the displacement of the piston of area *A* and *ΔV* the displaced volume of water. At the instant at which a volume of water is transferred by the effect of the pressure, a gradient of water chemical potential is created because solutes concentrate in the compartment at which pressure is applied and dilute in the opposite one, producing a flux of water from the diluted to the concentrated solution. This opposes to the volume decrease imposed by pressure. Now, let us consider the compartment on the left filled with a volume of concentration *C* of the same solute and the compartment on the right filled with the same volume of water. This will produce a flux of water from right to left increasing the volume of the concentrated solution, generating a pressure that will produce a flux of water from left to right.

The treatment of TPI has been found very suitable to explain the permeation processes in lipid bilayers [25,26]. The system is described by volume flux driven by differences in pressure between the inner and the outer compartment due to an osmotic gradient and by a diffusional flux of water coupled to the permeation of a solute across the membrane.

The whole system can be described by these equations [25, 26]

$$\begin{array}{l} J_{v}=L_{pp}\Delta P+L_{pD}\Delta C\\ J_{D}=L_{Dp}\Delta P+L_{DD}\Delta C \end{array}$$ (3)

In a vesicle, the difference in pressure is set by the difference in the osmotic concentration of solute given by Π = *RT*Δ*C*. Thus, *J*_*v*_ is the volume of water flux driven by the osmotic gradient **(**Π**)** and coupled to the diffusion of solute across the membrane. *J*_*D*_ is the diffusion of water and solute driven by the difference of pressure and that corresponding to the difference of solute concentration. The coefficient *L*_*pp*_ corresponds to the water mobility, *L*_*DD*_ the diffusional coefficient the membrane and *L*_*pD*_ and *L*_*Dp*_ are crossed coefficient coupling the two fluxes.

The permeation of non-electrolytes through lipid bilayers according to the concepts of irreversible thermodynamics show a strong interaction between the solute and water that implies a number of water molecules copermeating with each molecule of solute. The number of copermeating water molecules is independent of the nature of the permeant which denotes that the effect is due to osmotic forces. This formalism can be extended or analogously reproduced in lipid monolayers if it is taken as an open bidimensional solution that may exchange water with the subphase.

Thus, the resulting formalism is:

$$\begin{array}{ll} J_{a} & =L_{\Pi \Pi }\Delta \pi +L_{D\pi }\Delta C\\ J_{D} & =L_{D\Pi }\Delta \pi +L_{DD}\Delta C \end{array}$$ (4)

where *J*_*a*_ is the area change by the surface pressure π (note that lower and higher case the osmotic pressure in Eq 3, i.e. the surface applied on the barrier, and by the difference in chemical potential of water (equivalent to the solute) in the monolayer. *J*_*D*_ is the diffusional flux produced by the change in monolayer surface pressure due to water flux driven by the concentration of solute between the interphase and the bulk of the subphase. In this analysis, it becomes clear that thermodynamically speaking the monolayer is equivalent to the bilayer. The difference is that in bilayers the flux of solution is expressed as a volume change of the vesicles and in monolayers as an area change. By extension, the same approach should apply for the surface pressure area lipid process. This is analyzed in the next section.

## Results

The surface pressure *π*arising in monolayers of insoluble amphiphilic compounds has been interpreted as due to the bombardment of the barrier by the lipid molecules that have bidimensional translational energy, analogous to that of the gas pressure on the container wall. This is the ideal approach disregarding lipid-water interaction; that is absence of frictional work with the subphase when lipids are pushed in. According to Adamson and Gast, [27] another interpretation consists in regarding the barrier as a semipermeable membrane through which water may pass (in fact go around it) but lipids cannot go through. The monolayer can be considered as a concentrated solution having an osmotic pressure, which exerts a force on the membrane (barrier). When the lipid interphase is considered as a bidimensional gas the system is a closed one and is inconsistent to explain membrane response to solutes in the subphase. The second approach considers the interphase in an analogous way to a solution of a non-permeant solute (the lipid head groups immersed in water) [28].

The formalism of thermodynamic of irreversible process (TIP) [24] has not been so far developed for lipid monolayers considered as a bidimensional open system. TIP is based on the consideration that a system is subjected to several forces and equilibrium is reached when all of them are zero. Therefore, it is important to define the intensive forces participating in the system and the consequence that they may have on the variation of extensive properties such as area and number of molecules. The different experimental strategies indicate several forces that may act on different systems. For example, an osmotic gradient (difference in chemical potential of water between the inner and the outer solution across the membrane) produces volume changes in a cell or a close lipid vesicle. The osmotic gradient is the conjugated force of the volume change. However, it concomitantly affects the area of the lipid membrane, thus introducing cross mechanical phenomena. On the other hand, area changes can be induced in the monolayer by an external mechanical force applied on it. In this case, compression or expansion would cause changes in the water activity of the lipid interphase due to the changes in the lipid surface excess, thus promoting a water flux from and to the monolayer and the water bulk, respectively. Both processes are coupled and have cross-linked consequences.

TIP provides a way to give a rigorous frame to these crossed phenomena because it allows considering all the forces acting on the systems in a coupled way. Let us transfer the above reasoning to a lipid monolayer spread on water. For this, the monolayer is defined as a film of area A and a thickness δ. The compression and expansion of a monolayer can be done mechanically varying with an external barrier the area available for the lipids in the surface, as the piston in a volume of gas. On this base, a similar reasoning can be made if the barrier is considered as a semipermeable membrane and the non-permeant solutes are the lipids. In the scheme of Fig 3, when the barrier is moved to the left (red arrow), lipids concentrate on the surface producing an increase in the excess surface concentration of lipids (Γ_L_).

When area decreases the thickness increases and viceversa. Thus, the interrelation between area (A) and thickness (δ) implies that they compensate each other to maintain the volume constant, i.e. *V* = *Aδ* = *K* or *A* = *K*/*δ* and *dA* ≈ −*dδ*, where K is a constant.

In this case, the mechanical work done on the surface is,

$$W=f\Delta l=\left(\frac{f}{l}\right)\Delta A=\gamma \Delta A$$ (5)

where *γ* is the surface tension and *ΔA* the area change. As the work done by the barrier is against the pressure of the lipids the difference of the work in pure water and on the surface with lipids to create a similar area *ΔA* is

$$W=\left(\gamma^{0}-\gamma \right)\Delta A=\pi \Delta A$$ (6)

where π is the surface pressure. Thus, according to Eq 1, when the lateral pressure is exerted on the barrier, the area decreases at a certain rate (*J*_*a*_ = −*dA*/*dt*) at expense of π increase.

$$J_{a}=-l_{LL}\pi$$ (7)

This equation considers that the force (π) applied to the barrier to compress the monolayer is done by the friction of lipid with lipids, inverse to the coefficient *l*_*LL*_.

**Fig 4:**
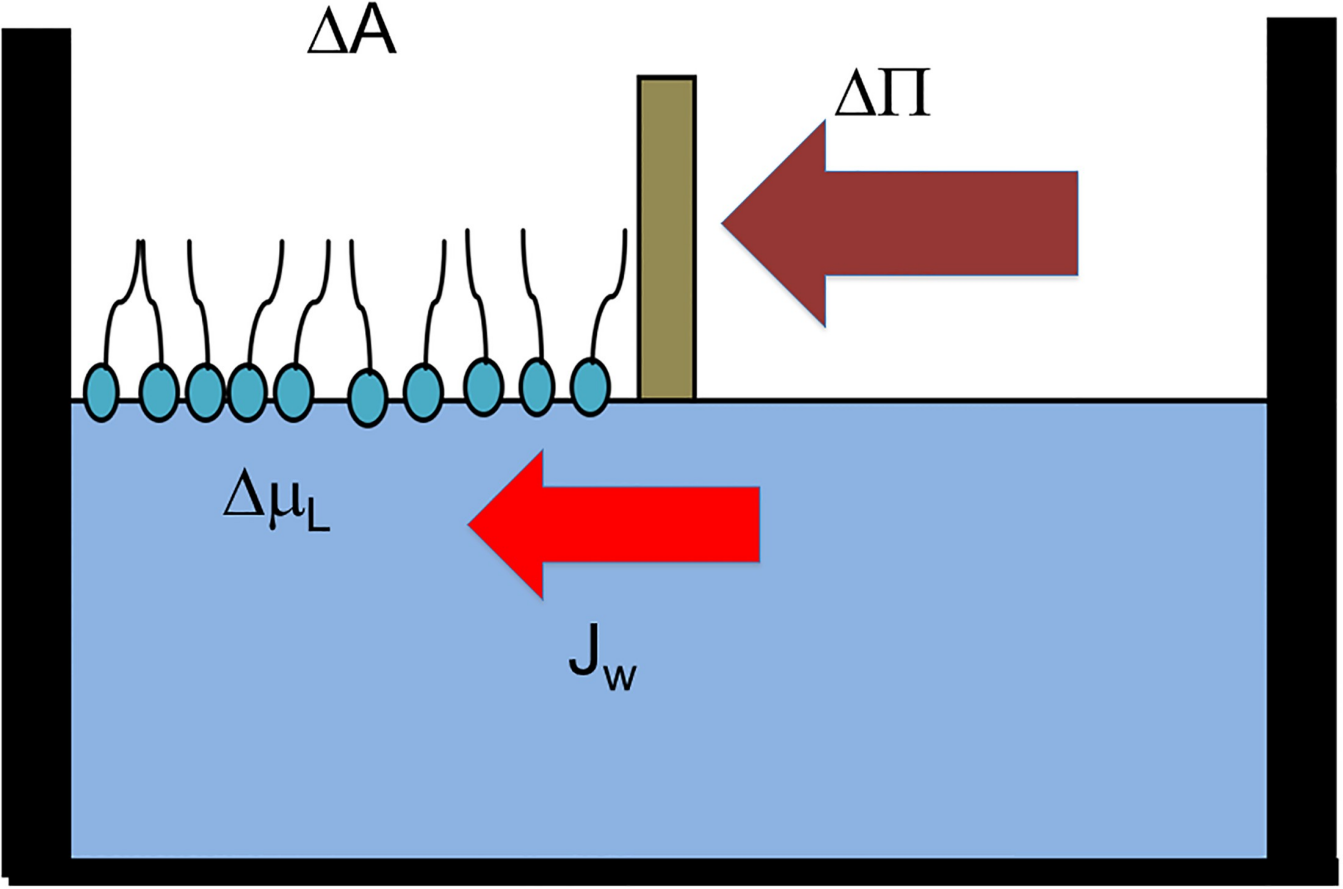
Schematic representation of the compression of a lipid monolayer by a mobile barrier on the surface of a water solution. Horizontal violet arrow represents the surface pressure that produces area changes by the compression. Vertical red arrow represents the water outflux from the interphase upon compession. The increase in surface concentration of lipids at the surface of the left-hand compartment consequently decreases the chemical potential of water. Vertical violet arrow corresponds to the water influx to the interphase due to the difference in the chemical potential of water between bulk and the interphase. Horizontal red arrow is the expansion (area increase) produced by the water influx. If expansion is produced mechanically the area increased induces the water influx.

On the other hand, the lipid excess (*Γ*_*L*_) increases as a result of the amount of lipids in relation to water area. This change in *Γ*_*L*_ is equivalent to a decrease in free water surface. In consequence, a gradient of the chemical potential of water between the surface in contact with lipids and the lipid-free water surface is produced that results in a diffusional flux of water (*J*_*D*_) from the bulk solution to the surface of lipids (vertical violet arrow in Fig 4) that tends to increase the area per lipid. This can be written as:

$$J_{D}=-l_{ww}RT\Delta \Gamma_{L}$$ (8)

Where *l*_*ww*_ represents the inverse of the resistance to flux due to the water-water interactions, with T absolute temperature and R the gas constant. Reciprocally, if lipids are added at an initial area on only one side, a difference in chemical potential will also operate resulting in a diffusion of water from the compartment without lipids to that with lipids, thus increasing the surface pressure and moving the barrier to the right (horizontal, violet arrow in Fig 4). Thus, the increase in area is given by

$$J_{a}=l_{Lw}\Delta \Gamma_{L}$$ (9)

In this case, *l*_*Lw*_ stands for the inverse of the friction of lipids in water. The decrease in area due to pressure extrudes water from the interphase (water outflux, vertical red arrow, Fig 4 and thus, the water flux from the interphase to the bulk, which is opposite to that driven by the difference in the chemical potential between the interphase and the bulk.

Thus,

$$J_{D}=l_{wL}\pi$$ (10)

Now, *l*_*wL*_ represents the inverse of the resistance to flux of water in lipids. Therefore, the total flux of water is

$$J_{D}=-l_{ww}RT\Delta \Gamma_{L}+l_{wL}\pi$$ (11)

and the area variation

$$J_{a}=l_{Lw}RT\Delta \Gamma_{L}-l_{LL}\pi$$ (12)

As

$$J_{D}=-\frac{1}{c_{w}}\frac{dn_{w}}{dt}=a_{L}\frac{dn_{w}}{dt}=\Gamma_{L}\frac{dn_{w}}{dt}=J_{L}=J_{a}$$ (13)

Where *dn*_*w*_/*dt* is the molar water flux, *c*_*w*_: the water concentration and *a*_*L*_: the molar area of the lipid. *J*_*L*_ the lipid flux is equivalent to the change of area *J*_*a*_.

From where, considering Eqs (11) and (12)

$$\pi =\frac{\left(l_{ww}+l_{Lw}\right)}{\left(l_{LL}+l_{wL}\right)}RT\Gamma_{L}$$ (14)

Considering that

$$\Gamma_{L}=\frac{n_{L}}{A}$$ (15)

when *l*_*ww*_ = *l*_*LL*_ and *l*_*wL*_ = *l*_*Lw*_ = 0 Eq 14 reduces to the ideal case.

$$\pi A=n_{L}RT$$ (16)

which is the equation of state corresponding to an ideal gaseous bidimensional system The null cross coefficients indicate that there are no interactions between water and lipids of the monolayer. In addition, *l*_*ww*_ = *l*_*LL*_, because in an ideal approach lipid particles and water particles are indistinguishable. On the other hand, Eq 14 can be written in terms of the molecular area *a*_*L*_ = *A*/*n*_*L*_ as:

$$\pi =\left(k_{1}+k_{2}\right)\frac{RT}{a_{L}}$$ (17)

where

$$k_{1}=\frac{l_{ww}}{l_{LL}+l_{Lw}}\;\text{and}\;k_{2}=\frac{l_{wL}}{l_{LL}+l_{Lw}}$$ (18)

The adequate selection of the *l*_ii_ coefficient allows to fit the experimental curves according to Eq 17 in different regions of the πvs Area per molecule isotherm.

Then, the only free parameter is *l*_*Lw*_. This can be parameterized as power laws with an exponent “m” to be determined.

$$l_{wL}=l_{wL}^{0}{\left(\frac{a_{0}}{a_{L}}\right)}^{m}$$ (19)

Where $l_{wL}^{0}$ is reference value and *a*_0_ = 42° Å^2^/molecule, has the dimension of area per molecule. This is included to keep the units. Renaming the constant parameters, the expression *k*_2_ takes the form

$$k_{2}=\frac{l_{wL}^{0}}{\left(l_{LL}+l_{Lw}\right)}{\left(\frac{42}{a_{L}}\right)}^{m}$$ (20)

Renaming the constant parameters, the expression 17 can be written as

$$\pi_{p}=\frac{k_{1}RT}{a_{L}}+\frac{k_{2}{}^{\prime }RT}{42}{\left(\frac{42}{a_{L}}\right)}^{p}$$ (21)

Where k_2_ has been redefined as:

$$k_{2}{}^{\prime }=\frac{l_{Lw}^{0}}{l_{LL}+l_{Lw}}$$ (22)

The exponent *p* = *m* + 1 is integer, and must be fitted to adjust the curvature.

**Fig 5:**
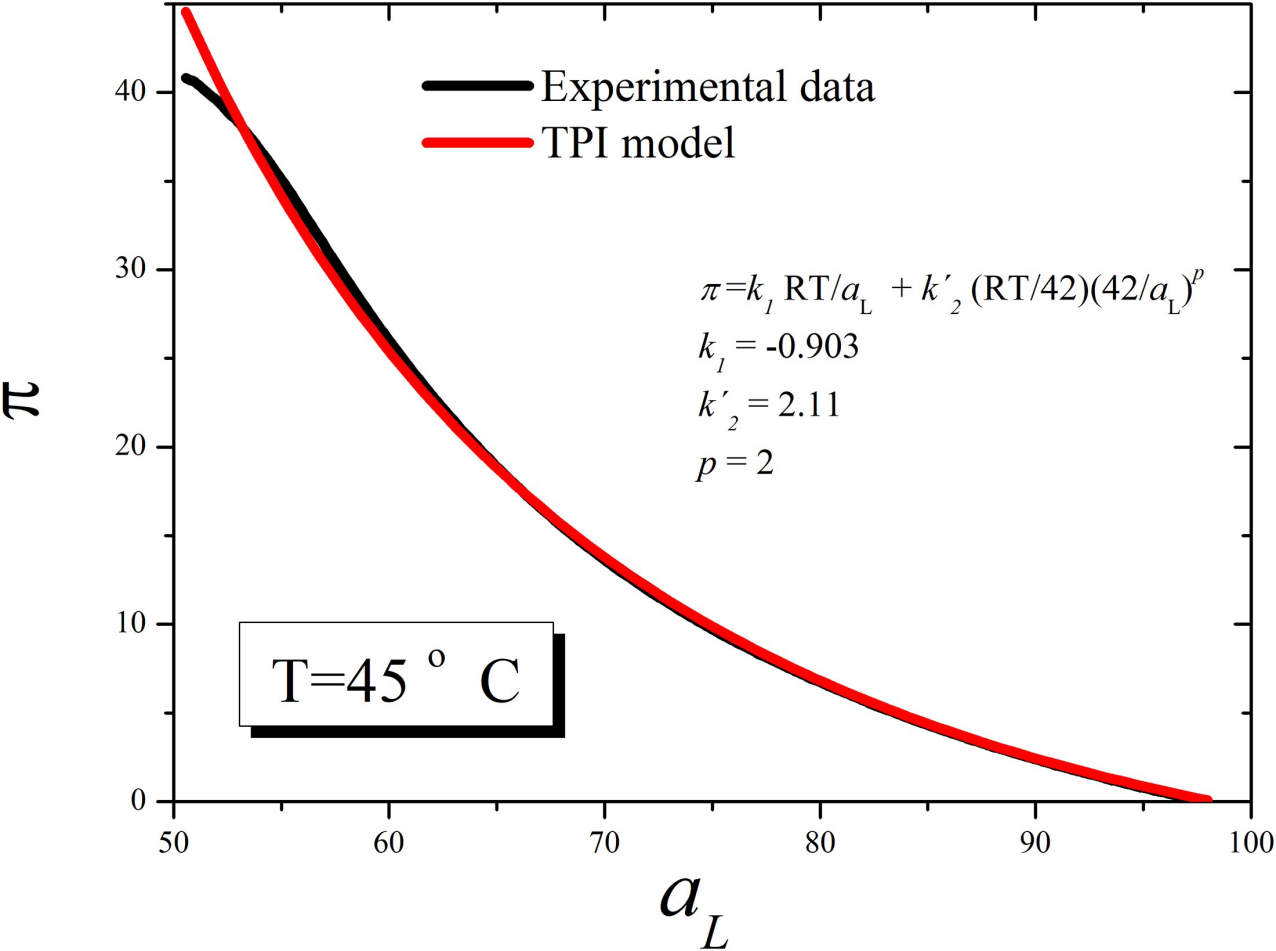
Surface pressures versus area per molecule for the TPI model at 45°C. Experimental (Black line) and calculated lipid curves for DPPC above the critical point according to Eq (21) at 45°C for a set of parameters corresponding to membranes.

Fig 5 shows the DPPC curves for *π*_*p*_ versus *a*_*L*_ for experimental data and the best fit for Eq (21) at *T* = 45°*C*.

**Fig 6:**
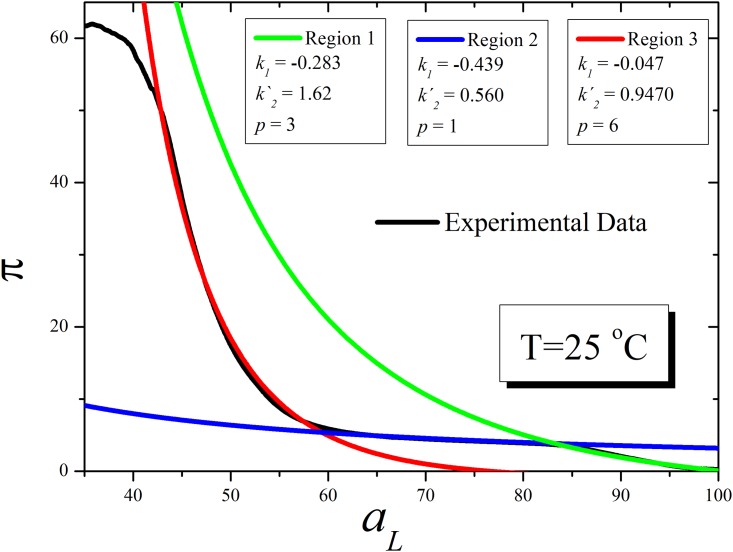
Surface pressures versus area per molecule fitted according to the TPI model at different region of the isotherm. Experimental curve as in Fig 1 (black line) were compared with calculated surface pressure vs area / molecule curves of DPPC considering the lipid monolayer as an open system according Eq (21) at 25°C. Green curve: region corresponding to the liquid expanded state. Red curve: region corresponding to the region of coexistence of liquid expanded and liquid condensed states. Blue curve: region corresponding to liquid condensed phase below the collapse. The parameters for the fitting in each region are summarized in Table 1.

Fig 6 shows the DPPC curves for *π* versus *a*_*L*_ for experimental data and several *p* values for Eq (21) at *T* = 25°*C*. As observed, the fitting of the experimental curves can be divided in three regions. Region I (green curve) corresponds to a monolayer in the liquid expanded phase. Region 2, the blue curve corresponding that represent the region of coexistence of the liquid expanded and liquid condensed states and the region 3 (red curve) to lipids in the liquid condensed curve. In each region there are specific indices that fit the experimental curves. Table 1 resumes all the fitted coefficients.

**Table 1:** Fitting parameters of π/A curves of Fig 5 for different regions of monolayer compression.

Region	Phase	*p*	k_1_	k´_2_
1	Liquid condensed	3	-0.28(3)	1.62(3)
2	Liquid expanded/liquid condensed coexistence	1	-0.43(2)	0.56(2)
3	Liquid expanded	6	-0.04(2)	0.94(2)

It is clear that different *k*_1_ and *k*_2_ coefficients correspond to each state for each state of the monolayer, which increases significantly from the liquid expanded to the liquid condensed state. This is reasonable since the lipid-lipid interaction and hydration of lipids is quite different in each state. At the liquid expanded state, the lipid-lipid interaction is minimum and the lipids are fully hydrated. Thus, lipids with smaller head groups should have greater *k* coefficient as denoted by Eq (21).

In the absence of lipids

$$\pi =\gamma^{0}-\gamma =0$$

which by definition should be *γ*^0^ = 72 dynes /cm or 7.2 mN/m for pure water, and γ the surface tension for a given lipid concentration in the interphase.

**Fig 7:**
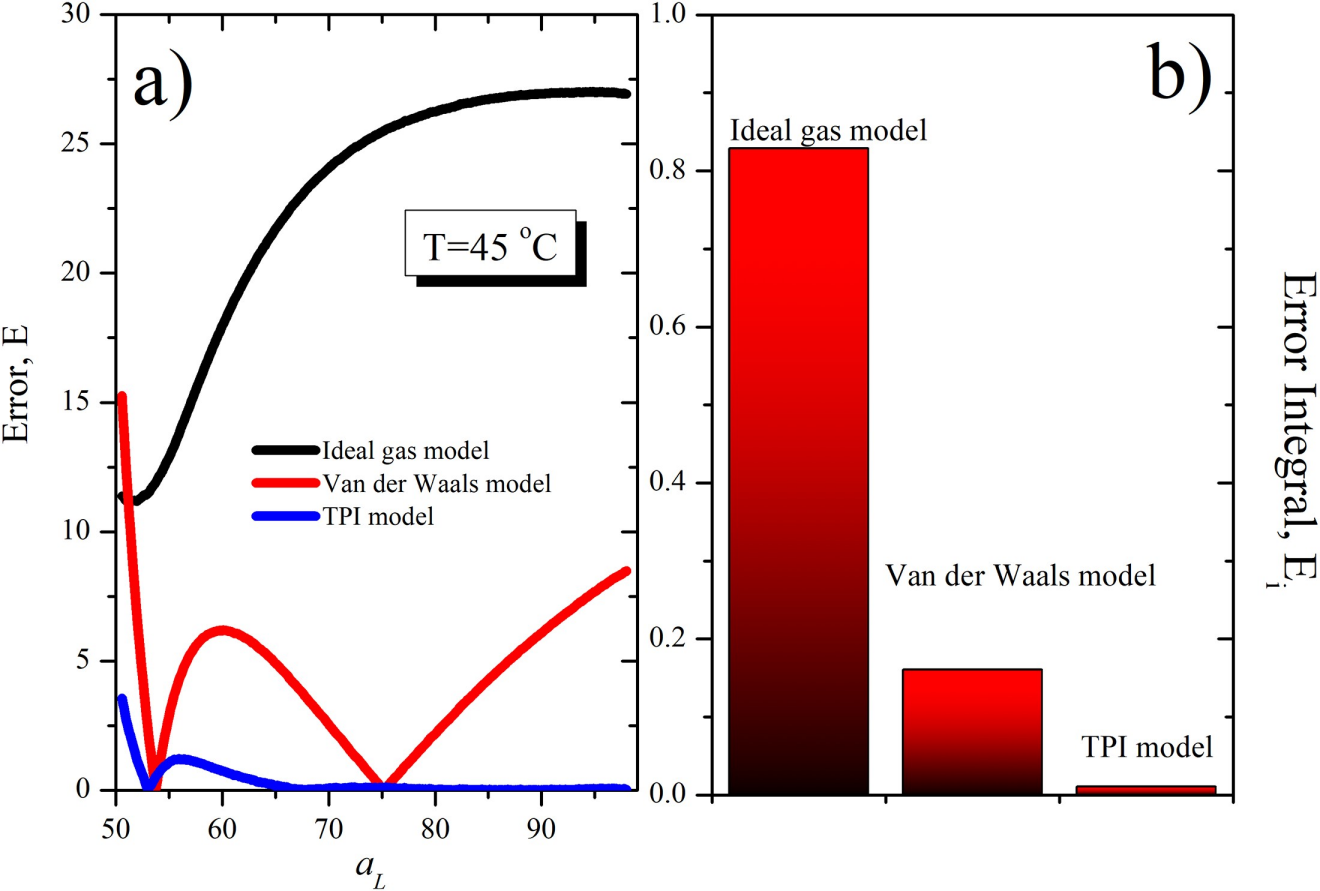
Errors at *T* = 45°*C* for ideal gas, van der Waals and TIP model. a) Error, E b) E_int_, integral error normalized. In both cases, TIP model has the smallest error.

Then, it is possible compare all approximations considering the next error expression:

$$E=\left|\pi_{\text{exp}}-\pi_{TPI}\right|$$ (23)

and the integral error normalized defined by

$$E_{\text{int}}=\int B\left|\pi_{Exp}-\pi_{TPI}\right|da_{L}$$ (24)

where B is a constant normalized. Fig 7(a) and 7(b) show these errors at *T* = 45°*C* for ideal gas, van der Waals and TIP model. According to this, throughout the range, TIP has the smallest error.

It is possible to relate the “*l*_*ii*_” coefficients with diffusion coefficients. Multiplying and dividing by RT, Eq (16) can be rewritten in terms of diffusion coefficient where the *l*_*ii*_ and *l*_*ij*_ represents the mobility coefficients. Thus,

$$k_{1}=\frac{D_{ww}}{D_{LL}+D_{Lw}}\;\text{and}\;k_{2}=\frac{D_{wL}}{D_{LL}+D_{Lw}}$$ (25)

For the purpose of this analysis, *D*_*ww*_ may be considered equal to pure water (10^−10^ to 10^−9^ m^2^/s) along the whole area changes in the surface pressure isotherms. This would correspond to the diffusion of water at high areas in which the contact between lipids is absent or low. At low areas, lipids touch each other through the hydration shells. However, it is reasonable that water would diffuse along the spaces in between those hydration shell of fixed water.

In turn, *D*_*LL*_ coefficient represents the lateral diffusion, in the plane of the membrane and varies according to the lipid-lipid interaction at each state of the monolayer. Thus, this coefficient may have different values at each portion of the π/A. That is, there is a *D*_LL_ value along the liquid expanded region; another along the liquid expanded-liquid condensed region (plateau) and a third one in the liquid condensed region. This is because the interaction depends on the state of the lipid acyl chains. In the liquid state D_LL_ is approximately 10^−8^ cm^2^/s above *Tc* approximately 10^−10^ cm^2^/s below Tc. [29, 30]. In the liquid condensed and liquid expanded coexistence region the *l* coefficient would be approximated by the semi sum of those in the fluid and the solid state.

With these considerations is possible use the fitting of the experimental data for obtain the unknown coefficients. At *T* = 45°*C* the next relations can be obtained

$$D_{Lw}^{0}=\frac{k{´}_{2}}{k_{1}}D_{ww}$$ (26)

$$D_{wL}^{0}=\frac{D_{ww}}{k_{1}}-D_{LL}$$ (27)

The values obtained are summarized in Table 2.

**Table 2:** Calculated coefficients for the diffusion of water in lipids (D_wL_) and the diffusion of lipid (with its hydration shell) in water (D_Lw_) in the different regions of the surface pressure / are per lipid isotherm.

*T* = 25°*C*(*<T*_*C*_)	*D*_*WL*_(*cm*^*2*^*/s*)	*D*^*0*^_*Lw*_ (*cm*^*2*^*/s*)
Liquid condensed	3.57x10^-6^	5.78x10^-6^
Liquid expanded/liquid condensed coexistence	2.32x10^-6^	1.30x10^-6^
Liquid expanded	2.5x10^-5^	2.3x10^-5^
*T* = 45°*C* (>*T*_*C*_)	*D*_*WL*_(*cm*^*2*^*/s*)	*D*^*0*^_*Lw*_ (*cm*^*2*^*/s*)
Liquid expanded	1.12x10^-6^	2.3x10^-6^

## Discussion

In a first approach, coefficient *l*_*LL*_ represents the molecular interaction between lipids considering two contributions: chain-chain and head group-head group interactions. According to the force field selected one may give different weights to the hydration levels and to the correlation of the two contributions [31, 32]. Thus, they should be taken as the friction of hydrated head groups in the bidimensional solution. Thus, it is reasonable that its value would depend on the state of the lipids since chain-chain and hydration are different in the liquid expanded and liquid condensed state. In this approach, the diffusion coefficient of water in bulk water is not altered in the presence of the lipids either in liquid condensed or in the liquid expanded state.

The water in lipid diffusion given by *l*_wL_ considers water surrounded by a lipid matrix. When the lipid matrix is condensed the hydration shell are in contact but there are spaces enough for water to diffuse between holes. In this context, the diffusion of water in between the lipid matrix can be considered equal to the diffusion in pure water. On the other hand, the l_LL_ coefficient will be dependent on temperature and changes sensible if the lipids are below or above the transition temperature. This means that l_LL_ would be different in the liquid expanded state, the liquid condensed state and in the region of coexistence of both states (see curve). Similarly, as the hydration varies in the different states, each portion of the curve will demand different values for the l_Lw_ coefficients. For this reason, the fitting was done for different regions of the isotherm as denoted in Fig 6.

## Conclusions

As stated above, TIP formalisms allow to introducing the coupling of area changes (lipid flux) and water fluxes. According to Eqs 11 and 12, changes in area can be expected to change the chemical potential of water at the interphase. For this reason, it is possible to extent this approach to other membrane systems such as bilayers in closed vesicles and further more in cells. This can be immediately done considering that changes in surface pressure by water activity can be achieved by osmosis, i.e. with the presence in the subphase of solutes excluded from the interphase. The osmotic stress can be produced in a closed vesicle by a gradient of osmolytes between the inner and the outer solution. In the case of vesicles in hypertonic solutions, water will be extruded from the bilayer decreasing the area per lipid, i.e. compressing both monolayers similar to the scheme in Fig 4. Thus, this formalism can be in principle applied to bilayers in closed vesicles. However, the compression may be larger than that needed to compress the lipids and may produce a collapse of the bilayer. This will result in regions of high curvature. The analysis presented in this work considers the compression of the monolayer and bilayers in a range below that at which curvature or deformations occurs.

As the formalism of TIP considers coupling of forces of different nature, the analysis of the monolayer behavior considers the conjugated force (the surface pressure change) that modifies the area/molecule and as a coupled phenomenon the water efflux and influx. Due to this consideration the formalism can also be applied to systems in which water chemical potential is the driving force. This is more precisely observed when vesicles surrounded by a bilayer are subjected to osmotic gradient. When vesicles swell in hypotonic solution the influx promotes an expansion of the membrane area, and viceversa in hypertonic solutions.

In conclusion, it has been demonstrated for the first time that lipid monolayers behave as an open system considering the exchange of water between the lipids and the bulk phase in the process of compression and expansion. It emerges from the application of the Thermodynamics of Irreversible Process that the area change driven by a lateral pressure is coupled to the change in water activity due to the surface lipid excess. With this formalism, a new physical insight takes relevance consisting in that changes are driven by the variation of the water-lipid interaction along the process.

Another important derivation of this formalism is that it can be reduced to ideal process by considering *l*_*LL*_ = *l*_*ww*_ and the crossed coefficient nulls. This is valid only at very low or very high areas. At intermediate areas, considering that membrane is composed by lipids and water, the surface pressure is a function of water activity as proposed by Defay Prigogine[19].

Finally, the parameters introducing changes in the phenomenological coefficients as a function of area allow to extent the model to a bidimensional solution of hydrated lipids. The coupling of compression and water activity derived by TIP in monolayers can be extended to the behavior of bilayers subjected to osmotic stresses. The water outflux/influx imposes compression or expansion, respectively, changing the area per lipid. In this case, caution should be taken because at some pressures, changes in curvature and mismatching may appear and this demands a much careful topological analysis.

## Supporting information

S1 File **Fig A**: Comparison of van der Waals gas with Surface pressure/ área isotherms of monolayers. **Fig. B**: The classical Surface pressure/área per lipid isotherm is generally described by different lipid organizations (right hand figure) **Fig. C**: Water-lipid (a) and lipid-water (b) interactions at high and low pressures.
(PDF)

## References

[1] Marsh D. Lateral pressure in membranes. Biochim. Biophys. Acta (BBA)-Reviews on Biomembranes, 1996, 1286, 183–223. 8982283 10.1016/s0304-4157(96)00009-3

[2] Feng SS, Interpretation of mechanochemical properties of lipid bilayer vesicles from the equation of state or pressure−area measurement of the monolayer at the air−water or oil−water interface, Langmuir, 1999, 15, 998–1010. 10.1021/la051216n 16519504

[3] MacDonald RC, Simon SA. Lipid monolayer states and their relationships to bilayers, Proc. Natl, Acad. Sci. USA, 1987; 84 4089–4093 3473494 10.1073/pnas.84.12.4089 PMC305028

[4] Rawicz W, Smith BA, McIntosh TJ, Simon SA, Evans E. Elasticity, Strength, and Water Permeability of Bilayers that Contain Raft Microdomain-Forming Lipids, Biophysical Journal, 2008, 94: 4725–4736. 18339739 10.1529/biophysj.107.121731 PMC2397373

[5] Evans EA, Skalak R. Mechanics and Thermodynamics of Biomembranes. CRC Press, Inc., Boca Raton, FL 1980.

[6] Jyoti A, Prokop RM, Neumann AW. Manifestation of the liquid-expanded/liquid-condensed phase transition of a dipalmitoylphosphatidyl choline monolayer at the air-water interface. Colloids and Surfaces B: Biointerfaces, 1997: 8,115–124.

[7] Dynarowicz-Łatkaa P, Dhanabalanb A, Oliveira ON Jr.. Modern physicochemical research on Langmuir monolayers, Advances in Colloid and Interface Science, 2001; 91, 221–293. 11392357 10.1016/s0001-8686(99)00034-2

[8] Gong K, Feng S-S, Go ML, Hsing Soew P. Effects of pH on the stability and compressibility of DPPC/cholesterol monolayers at the air–water interface, Colloids and Surfaces A: Physicochemical and Engineering Aspects 2002; 207, 113–125.

[9] Nagle JF. Theory of lipid monolayer and bilayer phase transitions: effect of headgroup interactions. The Journal of membrane biology, 1976; 27, 233–250. 940146 10.1007/BF01869138

[10] Disalvo EA, de Gier J. Contribution of aqueous interphases to the permeability barrier of lipid bilayer for non-electrolytes. Chem Phys. Lipids, 1983; 32, 39–47.

[11] Nagle JF, Tristram-Nagle S. Biochim. Biophys. Acta 2000; 1469, 159. 11063882 10.1016/s0304-4157(00)00016-2 PMC2747654

[12] Nickels JD, Katsaras J. Water and Lipid Bilayers, Chpt. 3 pp 45–68 in “Membrane Hydration: The role of water in the structure and function of biological membranes”, (ed. Disalvo E A.), Springer, Switzerland 2015

[13] Tristram-Nagle S. Use of X-Ray and Neutro Scattering methods with Volume measurements to determine lipid bilayer structure and number of water molecules /lipid, Chpt 2 pp. 17–44, in “Membrane Hydration: The role of water in the structure and function of biological membranes”, (ed. Disalvo E A.), Springer, Switzerland 2015 10.1007/978-3-319-19060-0_2 26438260

[14] Parsegian VA, Fuller N, Rand RP. Measured work of deformation and repulsion of lecithin bilayers. Proc. Natl. Acad. Sci. USA 1979; 76, 2750–2754. 288063 10.1073/pnas.76.6.2750 PMC383686

[15] Pfeiffer H., Hydration forces between lipid bilayers: A theoretical overview and a look on methods exploring dehydration, Chpt. 4, pp 69–104, “Membrane Hydration: The role of water in the structure and function of biological membranes”, (ed. Disalvo E A.), Springer, Switzerland 2015 10.1007/978-3-319-19060-0_4 26438262

[16] Reddy AS, Warshaviak DT, Chachisvilis M. Effect of membrane tension on the physical properties of DOPC lipid bilayer membrane. Biochim. Biophys. Acta, 2012: 1818(9) 2271–2281. 10.1016/j.bbamem.2012.05.006 22588133 PMC3574165

[17] Bouchet AM, Frías MA, Lairion F, Martini F, Almaleck H, Gordillo G, et al Structural and dynamical surface properties of phosphatidylethanolamine containing membranes. Biochim. Biophys. Acta Biomembranes, 2009; 1788, 918–925. 10.1016/j.bbamem.2009.02.012 19248762

[18] Jendrasiak GL, Hasty JH. The hydration of phospholipids. Biochim. Biophys. Acta 1974; 337, 79 91. 4474012 10.1016/0005-2760(74)90042-3

[19] Defay R., I. Prigogine Surface Tension and Adsorption, John Wiley & Sons: New York 1966.

[20] Disalvo E. A., Hollmann A, Semorile L, Martini M.F., Evaluation of the Defay-Prigogine model for the membrane interphase in relation to biological response in membrane-protein interactions. Biochim. Biophys. Acta (BBA)-Biomembranes, 2013; 1828, 1834–1839. 23567914 10.1016/j.bbamem.2013.03.026

[21] Chen D, Santore M M. Large effect of membrane tension on the fluid–solid phase transitions of two-component phosphatidylcholine vesicles, Proc. Natl. Acad. Sci, USA, 2014; 111 (1)179–184 10.1073/pnas.1314993111 24344297 PMC3890780

[22] Duncan SL, Larson RG. Comparing Experimental and Simulated Pressure-Area Isotherms for DPPC, Biophysical Journal. 2008; 94, 2965–2986. 18199666 10.1529/biophysj.107.114215 PMC2275714

[23] Alarcón LM, de los Angeles Frías M, Morini MA, Sierra MB, Appignanesi GA, Disalvo EA; Water populations in restricted environments of lipid membrane interphases, Eur. Phys. J. E. 2016; 39: 94 10.1140/epje/i2016-16094-5 27761781

[24] Katchalsky A, Curran P. Non Equilibrium Thermodynamics in Biophysics, Harvard University Press, Cambridge, MA, 1965.

[25] Van Zoelen EJJ, Blok MC, Stafleu GP, Lancée-Hermkens AMW, De Gier J. A molecular basis for an irreversible thermodynamic description on non-electrolyte permeation through lipid bilayers, Biochim.Biophys. Acta 1978; 320–334 10.1016/0005-2736(78)90270-5 687615

[26] van Zoelen EJJ, Henriques de Jesus C, de Jonge E, Mulder M, de Gier J. Non-electrolyte permeability as a tool for studying membrane fluidity. Biochim. Biophys. Acta, 1978; 335–347 10.1016/0005-2736(78)90271-7 687616

[27] Adamson AW, Gast AP Physical Chemistry of Surfaces, Wiley Interscience, (1997)

[28] Damodaran S. Water activity at interfaces and its role in regulation of interfacial enzymes: A hypothesis, Colloids and Surfaces B: Biointerfaces. 1998; 11, 231–237.

[29] Fahey PF, Webb WW. Lateral diffusion in phospholipid bilayer membranes and multilamellar liquid crystals. Biochemistry. 1978, 17(15):3046–3053. 698183 10.1021/bi00608a016

[30] Ladha S, Mackie A R, Harvey LJ, Clark DC, Lea EJ, Brullemans M, Duclohier H. Lateral Diffusion in Planar Lipid Bilayers: A Fluorescence Recovery after Photobleaching Investigation of Its Modulation by Lipid Composition, Cholesterol, or Alamethicin Content and Divalent Cations, Biophysical Journal 1996; 71:1364–1373. 8874012 10.1016/S0006-3495(96)79339-6 PMC1233605

[31] Disalvo EA, Lairion F, Martini F, Tymczyszyn E, Frías M, Almaleck H, Gordillo GJ. Structural and functional properties of hydration and confined water in membrane interfaces, Biochim. Biophys. Acta. 2008; 1778, 2655 10.1016/j.bbamem.2008.08.025 18834854

[32] Heimburg T. *Thermal biophysics of membranes*. John Wiley & Sons, Germany, 2008.

