# Supplementary material for: A new model for lipid monolayer and bilayers based on thermodynamics of irreversible processes
Source: PLoS One. 2019 Apr 4;14(4):e0212269. doi: 10.1371/journal.pone.0212269 (PMC6448890; doi:10.1371/journal.pone.0212269)
Supplement: S1 File — Fig A: Comparison of van der Waals gas with Surface pressure/ área isotherms of monolayers. Fig. B: The classical Surface pressure/área per lipid isotherm is generally described by different lipid organizations (right hand figure) Fig. C: Water-lipid (a) and lipid-water (b) interactions at high and low pressures. (PDF) [file pone.0212269.s001.pdf]

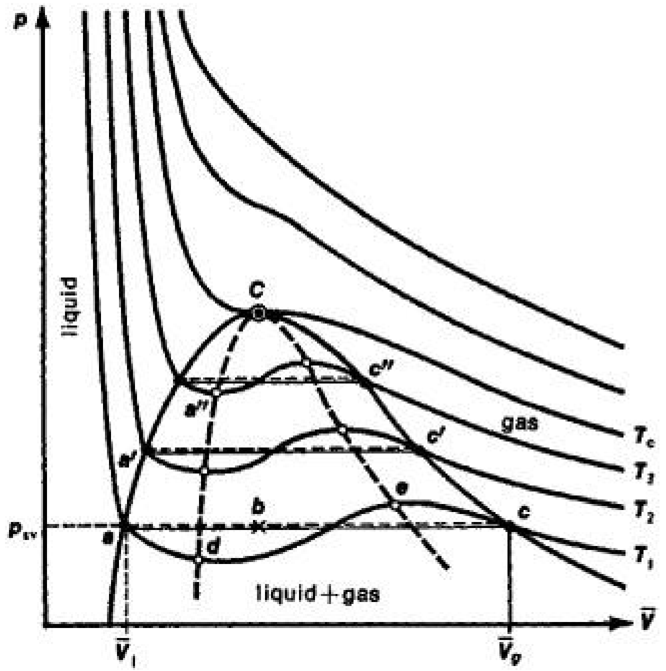

$$(p+\frac{a\cdot n^2}{V^2})(V-n\cdot b)=n\cdot R\cdot T$$

p	-	pressure
V	-	volume
T	-	temperature
R	-	gas constant
a, b	-	specific constants for each gas

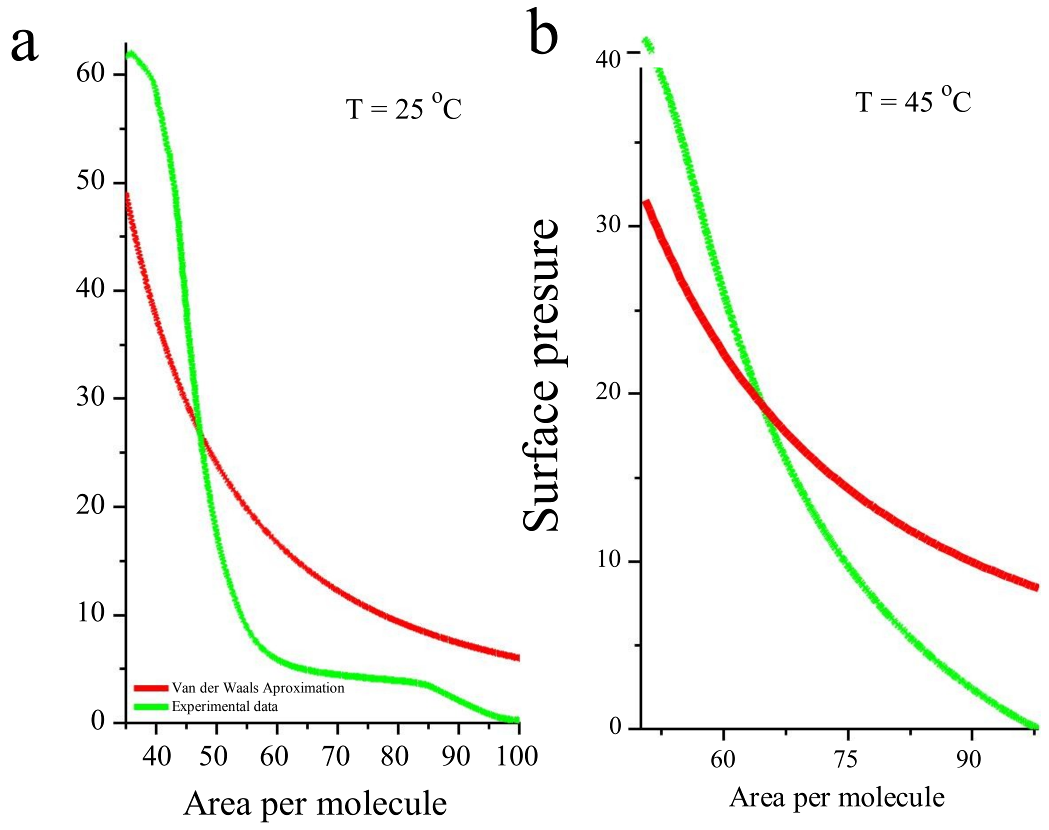

Although the trace of the experimental curve in monolayers (green curve) are simlar to a PV van der Waals gas (left hanf figure), fitting at 25C or 45C with a van der Waals equation neglecting wáter-lipid interactions does not reproduce the experimental data (red lines)

Fig. A: Comparisson van der Waals gas with Surface pressure/ área isotherms of monolayers.

$$\pi = (l_{WW} + l_{LW}/l_{LL} + l_{WL})\, RT\, \Gamma_L$$

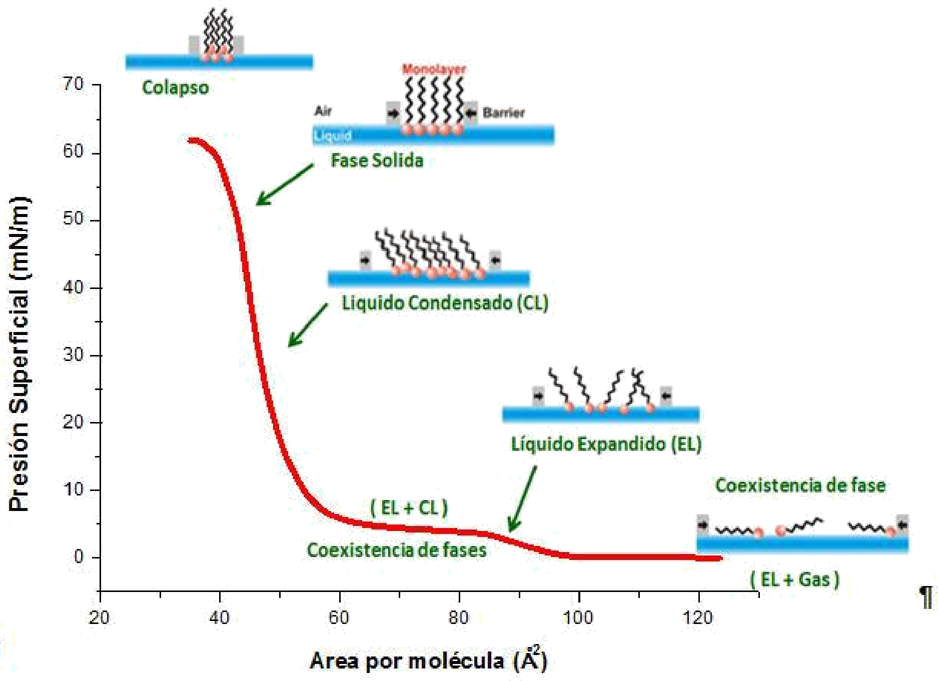

l_{WW} = *diffusion coefficient of pure wáter, Dw = RT lww*

l_{LL} = diffusion *coefficient of lipids in the membrane*

l_{WL} = diffusion of wáter in lipids.

L_{LW} = *diffusion of hydrated lipids difusión lípidos en agua con su capa de hidratación de los lípidos*

Fig. B: The classical Surface pressure/área per lipid isotherm is generally described by different lipid organizations (right hand fig.)

However, the thermodynamic description of Π vs Γ fitting data implies different coefficients of water lipid and lipid-water interaction along the process

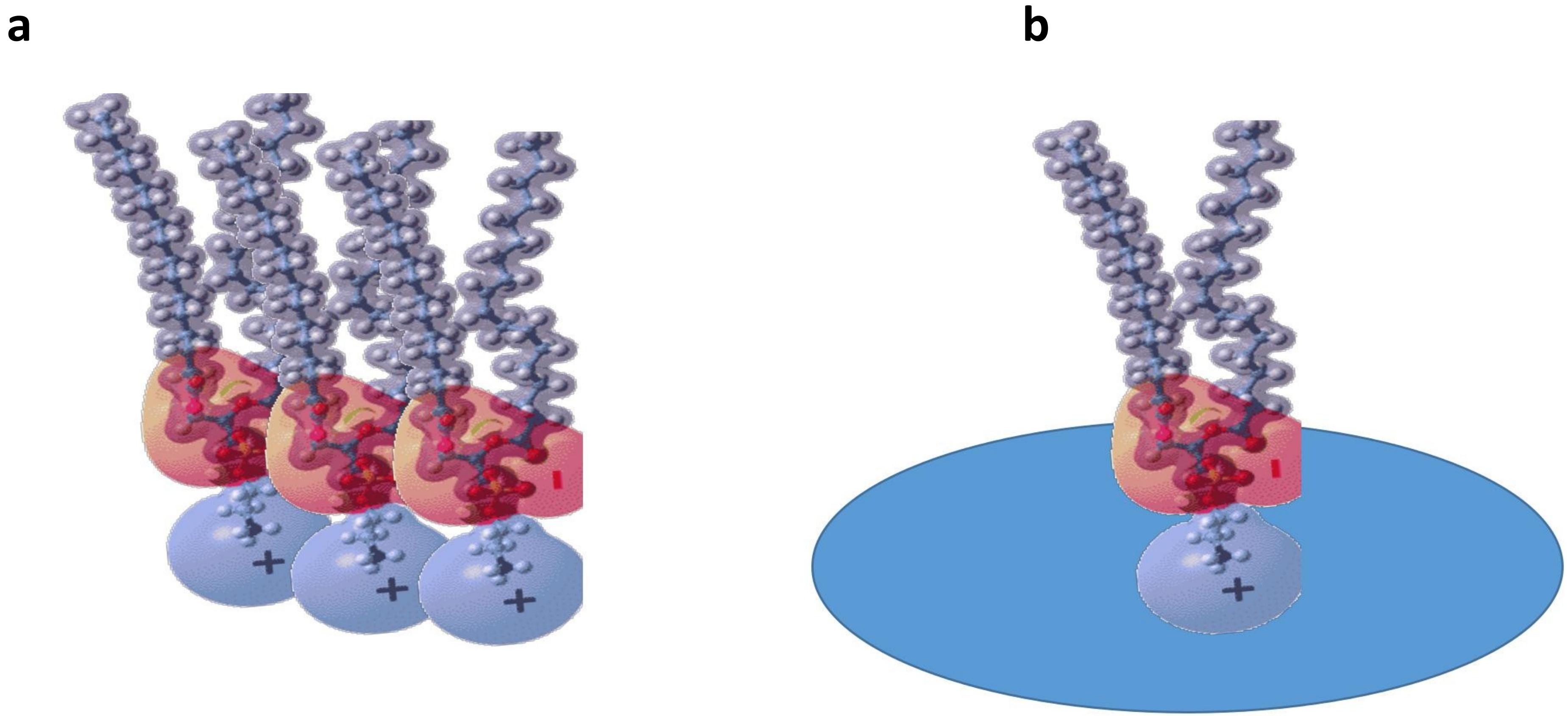

Fig. C: Water-lipid (a) and lipid-water (b) interactions at high and low pressures.
